# SLM Produced Porous Titanium Implant Improvements for Enhanced Vascularization and Osteoblast Seeding

**DOI:** 10.3390/ijms16047478

**Published:** 2015-04-02

**Authors:** Julia Matena, Svea Petersen, Matthias Gieseke, Andreas Kampmann, Michael Teske, Martin Beyerbach, Hugo Murua Escobar, Heinz Haferkamp, Nils-Claudius Gellrich, Ingo Nolte

**Affiliations:** 1 Small Animal Clinic, University of Veterinary Medicine Hannover, Foundation, D-30559 Hannover, Germany; E-Mails: julia.matena@tiho-hannover.de (J.M.); hugo.murua.escobar@med.uni-rostock.de (H.M.E.); 2 Institute for Biomedical Engineering, Rostock University Medical Center, D-18119 Rostock, Germany; E-Mails: svea.petersen@uni-rostock.de (S.P.); michael.teske@uni-rostock.de (M.T.); 3 Materials and Processes Department, Laser Zentrum Hannover e.V., D-30419 Hannover, Germany; E-Mail: m.gieseke@lzh.de; 4 Clinic for Cranio-Maxillo-Facial Surgery, Hannover Medical School, D-30625 Hannover, Germany; E-Mails: kampmann.andreas@mh-hannover.de (A.K.); gellrich.nils-claudius@mh-hannover.de (N.-C.G.); 5 Institute for Biometry, Epidemiology and Information Processing, University of Veterinary Medicine Hannover, Foundation, D-30559 Hannover, Germany; E-Mail: martin.beyerbach@tiho-hannover.de; 6 Division of Medicine Clinic III, Hematology, Oncology and Palliative Medicine, University of Rostock, D-18057 Rostock, Germany; 7 Institut fuer Werkstoffkunde, Leibniz Universitaet Hannover, D-30823 Hannover, Germany; E-Mail: haferkamp@iw.uni-hannover.de

**Keywords:** titanium implant, selective laser melting, polycaprolactone, VEGF, HMGB1, CXCL12, cross section, live cell imaging, osteoblast, cell migration

## Abstract

To improve well-known titanium implants, pores can be used for increasing bone formation and close bone-implant interface. Selective Laser Melting (SLM) enables the production of any geometry and was used for implant production with 250-µm pore size. The used pore size supports vessel ingrowth, as bone formation is strongly dependent on fast vascularization. Additionally, proangiogenic factors promote implant vascularization. To functionalize the titanium with proangiogenic factors, polycaprolactone (PCL) coating can be used. The following proangiogenic factors were examined: vascular endothelial growth factor (VEGF), high mobility group box 1 (HMGB1) and chemokine (C-X-C motif) ligand 12 (CXCL12). As different surfaces lead to different cell reactions, titanium and PCL coating were compared. The growing into the porous titanium structure of primary osteoblasts was examined by cross sections. Primary osteoblasts seeded on the different surfaces were compared using Live Cell Imaging (LCI). Cross sections showed cells had proliferated, but not migrated after seven days. Although the cell count was lower on titanium PCL implants in LCI, the cell count and cell spreading area development showed promising results for titanium PCL implants. HMGB1 showed the highest migration capacity for stimulating the endothelial cell line. Future perspective would be the incorporation of HMGB1 into PCL polymer for the realization of a slow factor release.

## 1. Introduction

Titanium is well established as implant material and can be manufactured accurately via Selective Laser Melting (SLM). SLM-made titanium constructions are commercially generated for this purpose (e.g., SLM Solutions GmbH, Luebeck, Germany) and allow the production of nearly any geometry [1]. In general, SLM can be described as wetting solidified material with liquid metal that is melted by laser radiation. Therefore, it is necessary that oxide layers are removed as they interfere with the wetting process [2]. Titanium is known for its large solubility for oxygen [3]. Therefore it has been suggested that oxides can be dissolved [4] and thus medical devices can be manufactured by SLM that provide an adequate surface quality [5]. Implant geometry is a key factor in bone modeling [6]. Especially porous implants support tight bone-implant interface. Different studies demonstrated an efficient bone ingrowth into porous titanium scaffolds [7,8], whereas the pore size examined for bone tissue engineering is very heterogeneous and varies from 20 to 1500 µm [9]. In general, porous titanium is well known as being stable and osteoconductive [8]. To achieve enhanced bone ingrowth, it is important that the cells are able to migrate into the structures of the porous implant. The cells that are located in the porous structure are in need of fast vascularization to achieve sufficient nutrition. Especially in large sized osseous defects, the early vascularization is prerequisite, as a long distance must be bridged for transporting nutrients, growth factors, and supporting gas exchange [10,11]. Geometry with 250 µm pore size is known to support angiogenesis [12].

Furthermore, improved vascularization can be approached by proangiogenic factors [13,14]. Using migration factors, survival of cells located in the scaffold can be improved. Vascular endothelial growth factor (VEGF) is well known as a proangiogenic factor, whereas high mobility group box 1 (HMGB1) has been discovered to also enhance angiogenesis [15,16]. Another factor that has been examined is chemokine (C-X-C motif) ligand 12 (CXCL12) in combination with HMGB1, where studies showed increasing chemotactic potency [17]. Achieving factor release, implant coating by biodegradable polymers with incorporated factors is common [18,19]. For this purpose, the polymer polycaprolactone (PCL) is applicable, as it is biocompatible, factors can be incorporated and it is already used for bone regeneration [20,21,22].

Herein, we ensured even PCL coating by Environmental Scanning Electron Microscopy (ESEM) and EDX measurements. *In vitro* results described an implant model based on an open porous titanium implant (later called titanium implant) in comparison with a PCL-coated titanium implant (later called titanium PCL implant). VEGF, HMGB1 and CXCL12 were examined using the endothelial cell line GM7373 in migration assays. To assess if cells are located in the porous structures of the implant, primary murine green fluorescent protein (GFP)-osteoblasts were seeded and examined by cross sections. Titanium implants and titanium PCL implants were compared using GFP–osteoblasts imaged in Live Cell Imaging (LCI). The cells were visualized directly on the implant surface to obtain a realistic interaction of cells with the implant material near to *in vivo* conditions.

## 2. Results

### 2.1. Manufacturing of Titanium Implants and Titanium Polycaprolactone (PCL) Implants

Titanium implants produced by SLM had a pore and strut geometry of 250 µm and total dimensions of 3.5 mm width, 3.5 mm depth and 1.25 mm height.

### 2.2. Characterization of PCL Coating on Porous Titanium Implants

**Figure 1:**
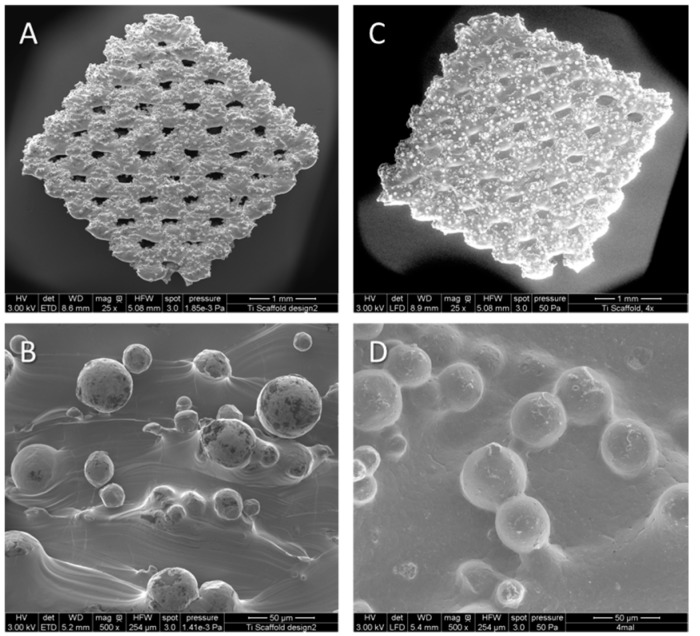
Representative Environmental Scanning Electron Microscopy (ESEM) micrographs of non-coated (**A**,**B**) and polycaprolactone (PCL)-coated (**C**,**D**) porous titanium scaffolds in overview and detail.

ESEM and EDX-measurements were performed to examine PCL coating on titanium implants. Surface coating of PCL coated implants could be visualized (Figure 1C,D). The atomic percentage of carbon and oxide determined by EDX measurements was clearly higher in coated implants in comparison to the non-coated titanium implants (Table 1).

**Table 1:** Surface composition of titanium implants and titanium PCL implants analyzed by EDX measurement. The relevant elements titanium, carbon and oxygen (Ti, C and O) described in atomic percent (At-%) are listed.

Scaffold Modification (At-%)	Titanium Implant	Titanium PCL Implant
Ti	70.09	3.04
C	4.68	74.87
O	0.87	21.41

### 2.3. Cross Sections Established of Titanium Implants

**Figure 2:**
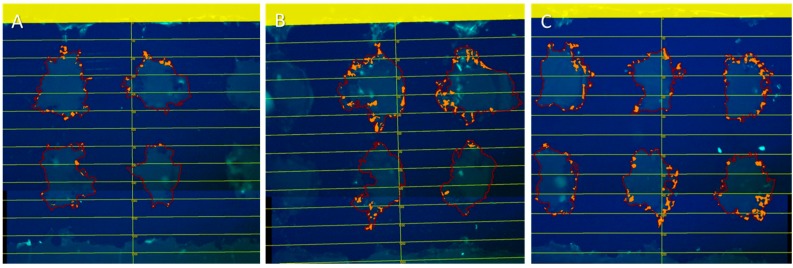
Cross section of titanium implant with green fluorescent protein (GFP)–osteoblasts settled for (**A**) one day; (**B**) three days; and (**C**) seven days. Cells were placed on the top of the implant (marked 0 µm). The distance between the horizontal lines is 100 µm. Pores are marked red and counted cells orange (Wimasis Image Analysis).

**Figure 3:**
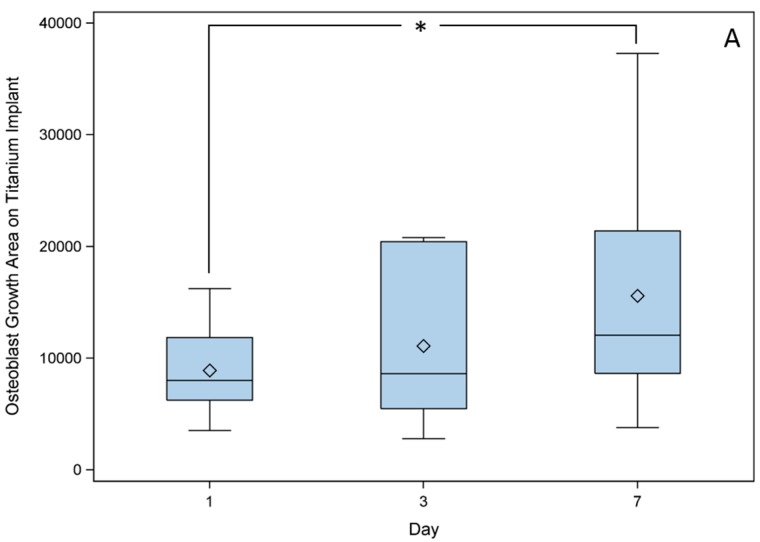

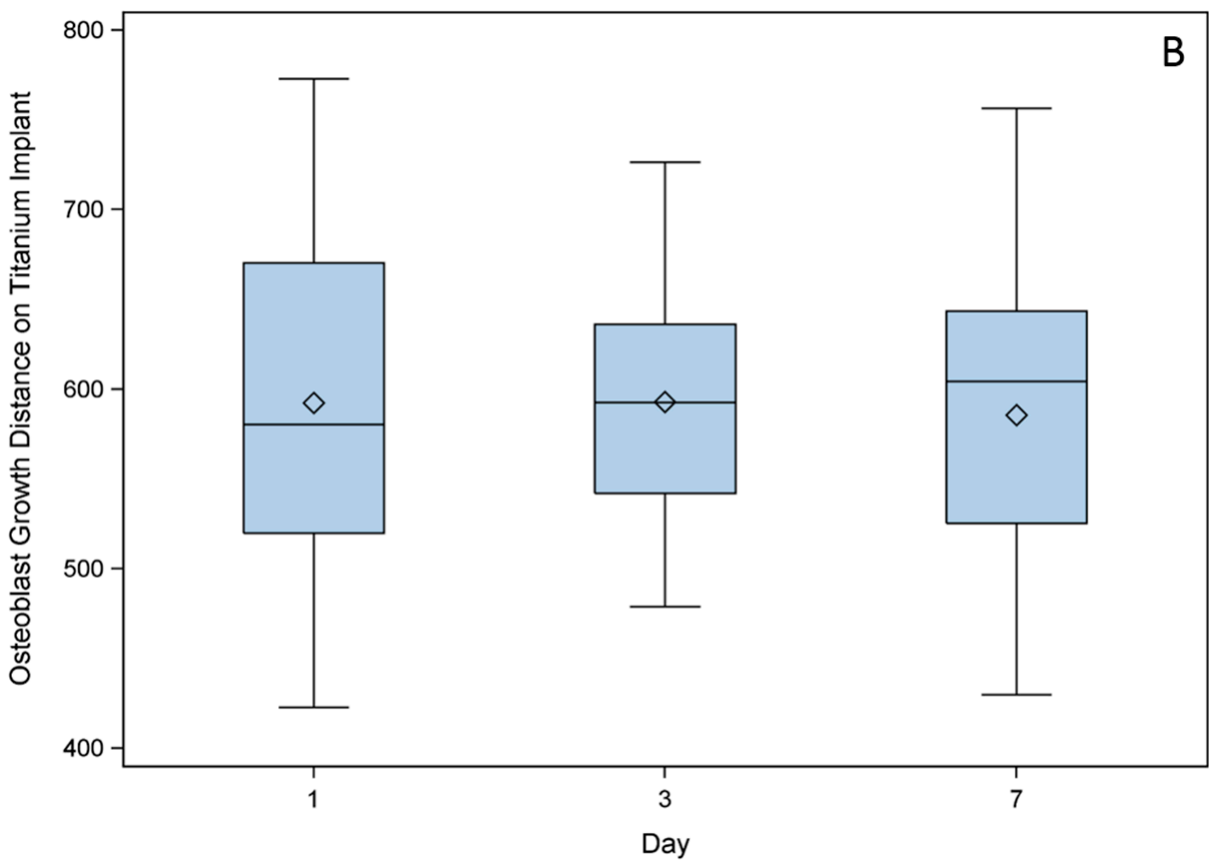
Cross sections were analyzed for changes in (**A**) total area of cell growth (µm^2^) and (**B**) growth distance (µm) of osteoblasts from top–bottom pores at different time points (day 1, day 3, day 7). Osteoblast growth area was significantly higher on day 7 in comparison with day 1, whereas osteoblast growth distance was nearly the same over the seven days. Statistical analysis was performed using the Global *F*-Test from the Analysis of Variance and Ryan–Einot–Gabriel–Welsh Multiple Range Test with *n* > 16 (* *p* < 0.05).

Cross sections of the titanium implants (Figure 2) showed that the cell amount in both the upper and lower pores increased over time and showed a significant difference between days 1 and 7. (Figure 3A). No increase in cell distance from the starting point towards deeper parts occurred (Figure 3B). Additionally, the cell amount between the lower and upper pore was similar during the seven days. Statistical analysis was performed using the Global *F*-Test from the Analysis of Variance and Ryan–Einot–Gabriel–Welsh Multiple Range Test (*p* < 0.05).

### 2.4. Migration Assays of GM7373 on Vascular Endothelial Growth Factor (VEGF), High Mobility Group Box 1 (HMGB1), Chemokine (C-X-C Motif) Ligand 12 (CXCL12)

**Figure 4:**
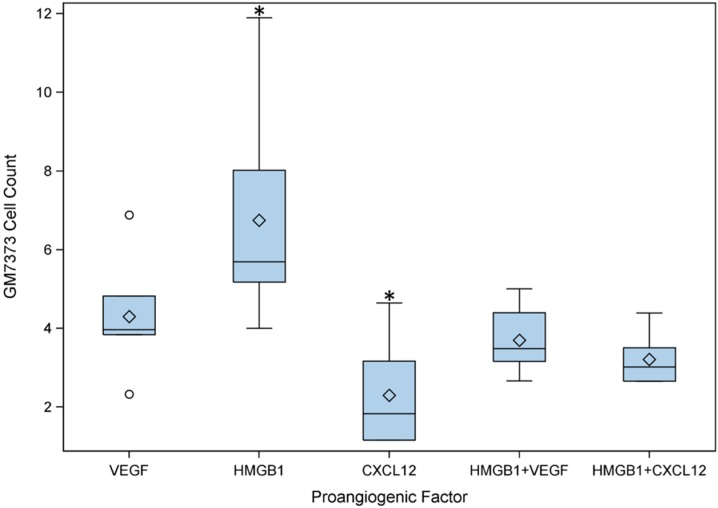
Comparison of chemotactic behavior of the endothelial cell line (GM7373) using vascular endothelial growth factor (VEGF), high mobility group box 1 (HMGB1), chemokine (C-X-C motif) ligand 12 (CXCL12) and combinations of HMGB1/CXCL12 and HMGB1/VEGF. For migration assays the negative control was set one and the other factors are its multiple. HMGB1 was more chemotactic than VEGF. CXCL12 induced lower migration of endothelial cells. Both factor combinations showed similar chemotaxis. Statistical test is two sample *t*–test with *n* = 6 (* *p* < 0.05).

The endothelial cell line showed the highest chemotaxis using HMGB1 (Figure 4). CXCL12 alone was less effective than VEGF. Combining a half a dose of HMGB1/CXCL12 and HMGB1/VEGF showed similar migration efficiency (Figure 4). To prove that CXCL12 supports migration in combination with HMGB1, additional assays were performed with different concentrations of HMGB1 and CXCL12. HMGB1 in the highest concentration (150 ng/mL) together with CXCL12 (30 ng/mL) showed significantly higher chemotaxis for GM7373 compared to the other combinations of HMGB1 and CXCL12. Two sample *t*-test was used as statistical test (*p* < 0.05).

### 2.5. Live Cell Imaging (LCI)

**Figure 5:**
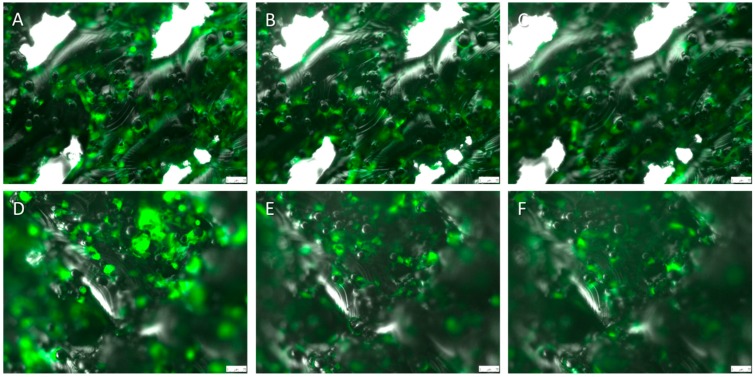
Titanium implant (90 degree angle and visible pore structure) after (**A**) 0 days; (**B**) three days; and (**C**) seven days and titanium PCL implant (45 degree angle and invisible pore structure) after (**D**) 0 days; (**E**) three days; and (**F**) seven days of GFP–osteoblast settling. (Scale bar: 75 µm)

**Figure 6:**
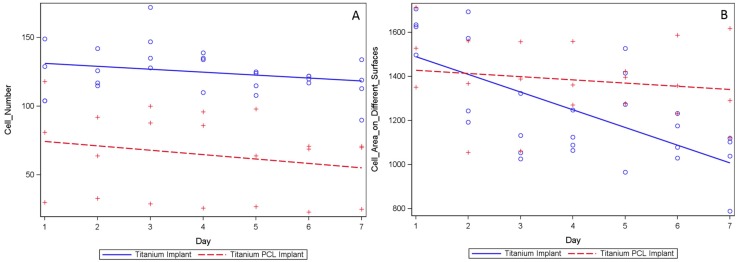
Evaluation of average GFP–osteoblast cell number changes (**A**) and cell spreading area (µm^2^/cell) changes (**B**) during seven days settled on titanium (the solid line represents the cell development on the titanium implant with the single time points: ○) and titanium PCL implants(the dotted line represents the cell development on the titanium PCL implant with the single time points: +). The statistical approach used is comparison of the two regression coefficients using an analysis of covariance with a test of the interaction between the two implant materials and time with *n* > 3 (*p* < 0.05). The regression curves showed no changes in the number of cells over seven days, however there was a difference in the cell spreading area.

Fluorescent osteoblasts seeded on the implants were tracked (Figure 5), cell numbers counted and cell size examined. Regression analysis illustrated changes in the cell number and shape over time while the two sample *t*-test compares cells at every single time point. Tracking cell number changes over time showed no differences between the two regression curves of titanium implants and titanium PCL implants (Figure 6A). Total cell numbers were significantly higher on titanium implants in comparison with titanium PCL implants on days 2, 3, 4 and 7. The cell spreading area was significantly higher for titanium PCL implants on day 4 (Figure 6B). Regression curves were different for the cell spreading area at the two implants; the titanium PCL implant showed higher values. (Statistic used: comparison of the two regression coefficients using an analysis of covariance with a test of the interaction between the two implant materials and time with *p* < 0.05).

## 3. Discussion

Implant failure addressing larger bone defects is often attributable to insufficient bone healing, mainly depending on insufficient vascularization. Bone replacement material should provide adequate mechanical properties being comparable to native bone in order to withstand the external forces. Aim of this study was to generate and evaluate a titanium implant structure characterized by a defined porous structure in order to provide a supportive feature facilitating vascularization. Additionally proangiogenic factors were analyzed to support a fast vessel ingrowth. As these factors can be integrated in PCL, PCL coating was evaluated. In order to generate these defined porous structures within the implants SLM was chosen as manufacturing option. In general, manufacturing of defined porous structures can be achieved by different rapid prototyping methods. Common manufacturing methods of porous titanium implants are SLM, Electron Beam Melting (EBM) and Laser Engineered Net Shaping (LENS) [23]. Raw material used within these methods is titanium powder and implants are manufactured with either laser beam (SLM and LENS) or electron beam (EBM) [24]. Murr *et al.* reported many advantages of SLM and EBM in contrast to conventional manufactured titanium implants, such as improved mechanical properties [25]. Both the SLM and LENS process achieved similar strength, surface roughness and accuracy [26]. Despite of the manufacturing method, all porous titanium implants showed good biocompatibility [27].

To ensure cells are able to seed in the inner pore regions osteoblasts were seeded on the titanium implant, as they are essential for bone modeling. In general imaging and thus tracking of cells in the porous implant structures is challenging as commonly the materials are not transparent impeding visualization based analyses. Consequently, commonly used employed cell counting assays cannot detect cell location [28], while histology can determine cell count and location at once [29]. In this study fluorescent GFP–osteoblasts could be tracked in the porous structure analyzing cross sections. The cell surface area, which represents the number of cells, increased during the seven days of cell settling. The increased measured cell surface area is a value describing cell proliferation. Consequently, the results indicate that osteoblasts could proliferate into the pores. Furthermore, the proliferation effects are dependent on sufficient nutrition of the ingrowing cells. Thus the selected pore size allows sufficient media exchange to keep the cells vital. The chosen small pore size of 250 µm allowed high cell seeding inside the implant. An earlier study reported higher cell counts even for 500 µm pore size in comparison with 1000 µm pore size of SLM made titanium implants [6]. This difference in size effect was discussed to be associated to lower implant permeability allowing more cells to become attached, resulting in an increased surface area [6]. Hence, the small pore size is supposed to enhance cell attachment. The SLM process generates pores that are regularly manufactured, allowing cells to spread easily through the whole scaffold. In contrast, implants generated by powder metallurgy provide pores with irregular and undefined pore geometries. In such structures the cells are only able to seed on the implant surface when cultivated in a static medium system [29]. Larger scaffolds were herein not examined, thus it is not known how deep cells could be located in the scaffold. Despite the fact that the used cells were able to seed in the porous structures, a direct migration could not be observed. A more efficient cell seeding could be potentially achieved by using migration factors. In general, directed cell migration into the implant could lead to improved bone modeling [30].

Aiming at a perspective improved implant vascularization, different proangiogenic factors were evaluated. Large defects require large implant structures, accordingly fast vessel ingrowth is key to support osseogenesis [31]. In this study migration assays were performed using the endothelial cell line GM7373. Endothelial cells already showed potent chemotaxis response to HMGB1 in an earlier study [32]. In fact, HMGB1 was more effective concerning GM7373 migration efficiency than the “gold standard” reference factor VEGF [33]. The observed potent chemotactic effect of HMGB1 compared to VEGF is of major value, as VEGF is one of the most commonly used proangiogenic factors [34,35]. Consequently the next step would be the evaluation of migration assays with primary endothelial cells and further cell types that are commonly present in bone tissue such as osteoblasts.

CXCL12 has recently been focused as a chemotactic migration factor. Combining HMGB1 and CXCL12 should lead to improved chemotaxis, as recent studies showed that the factors were highly linked concerning mesoangioblasts, monocytes, macrophages and fibroblasts [17,36,37]. Schiraldi *et al.* demonstrated that HMGB1 and CXCL12 bind as a heterocomplex to the CXCR4 receptor [37]. Until now, little has been known about migration of endothelial cells as respond to CXCL12 stimulation. Herein it was demonstrated that the combination of HMGB1 and CXCL12 showed the same chemotactic efficiency for GM7373 compared to a HMGB1 and VEGF combination.

Chemotactic factors as the herein evaluated can be incorporated into polymers on surfaces of the implant material. Thereby a directed release over time can be achieved leading to an enhanced factor concentration into the area of interest [38,39]. EDX and ESEM analyses showed in this study complete surface covering of PCL on the coated titanium implants. Consequently, the herein employed dipping process is sufficient to achieve the desired titanium implant functionalization as the implant pores remained open after the coating. This is highly important to maintain the desired open porous structure and to enable vessel and bone ingrowth. As well implant functionalization with factors in the inner pore structure could be enabled.

However, polymer coating itself changes surface characteristics of the coated implant material and thus could result in different cell behavior. In order to evaluate a polymer induced effect of cellular seeding capacity surfaces with and without polymer coating were comparatively analyzed. LCI of both implant types allow the analyses of cellular parameters as morphology, cell count, and viability simultaneously. Thereby defined implant sections were settled with cells and observed during the seven days monitoring the cellular behavior on the examined surface (Supplementary Video S1 and S2). This methodical approach provides a unique possibility for examining cell behavior of opaque material. However, the commonly used assays for cell visualization on implant materials require the fixing of the cells prior to imaging, allowing only a single time point analyses without the possibility to monitor cell behavior over time [40,41]. The results herein showed that the total cell number was significantly higher on the titanium implant structures when compared to the titanium PCL implant. The observed lower cell numbers on titanium PCL implants could be lower due to the higher hydrophobic values of the PCL [42]. When cells settle for the first time, only few proteins attach themselves to the surface and thus fewer cells are able to attach themselves. After a while extracellular matrix (ECM) molecules can bind to the PCL and cells are able to attach equally well to the titanium implant [43].

These results suggests that titanium PCL implants are not suitable for osteoblasts, but *in vivo* studies already showed good properties for bone modeling [44]. When looking at the cell spreading area we do have good results for titanium PCL implant. Furthermore, the development of cell numbers over seven days is similar for both implants. These results show that cell counting of single time points can lead to different results. The cell spreading area and the development of cell numbers can be a helpful addition for cell evaluation on implants.

HMGB1, showing highest chemotactic potential, incorporated in PCL for porous titanium implant coating could be used in future research.

## 4. Experimental Section

### 4.1. Manufacturing Titanium Implant

For the titanium implant a TiAl6V4 titanium alloy was used. The implants were manufactured by SLM Solutions GmbH, Lübeck, Germany, with an SLM 280HL Selective Laser Melting system. Therefore a standard parameter set was taken using a laser power of 275 W, a scan speed of 805 W and a hatch distance of 120 µm for the core volume. The outer contour was produced using a laser power of 100 W and a scan speed of 350 mm/s.

### 4.2. Dip-Coating Process for Application of Polymeric Coatings to Porous Titanium Scaffolds

For implant coating a manual dip-coating process was established. First titanium implants were washed in isopropanol. For each implant, 2 mL of polymer solution was filled in the dipping tanks. Concentration was 0.4% of PCL. After each dipping process intermediate drying for 10 min at 23 ± 2 °C was performed. This was repeated six times. Finally the titanium PCL implants were dried in a vacuum at 40 °C for 24 h.

### 4.3. Scanning Electron Microscopy and EDX Measurements

To examine complete surface covering of the polymer coating, environmental scanning electron microscopy (Quanta FEG 250, FEI, Eindhoven, The Netherlands) equipped with an energy-dispersive X-ray (EDX) analysis unit was used. After fixing the titanium PCL implants and the titanium implants, the scanning electron micrographs were performed at 50 Pa pressure, moisturized atmosphere and an accelerating voltage of 10 kV. EDX measurements were performed at the beam entrance of the electron microscope. Titanium and carbon were determined by analyzing the spectra of the fibers bombarded with electrons.

### 4.4. Cell Culture

#### 4.4.1. Greenfluorescent Protein (GFP)–Osteoblast Isolation

Cell isolation was performed using adult GFP*C57Bl6 mice as previously described [45]. After mincing the bone of the calvarias into small pieces, 200 U/mL collagenase II (Cell Systems, Troisdorf, Germany) in Hank’s medium (HBSS, PAA Laboratories GmbH, Pasching, Austria) was used. Five milliliters collagenase solution was used for calvarias of ten mice. Digestion took place five times for 10 min at 37 °C. The supernatant of the final three steps was centrifuged (1200 rpm, 7 min). After washing the pellet twice with culture medium, cells were placed in culture plates and incubated at 37 °C. The medium used was DMEM (Biochrom AG, Berlin, Germany) with the addition of 10% fetal calf serum (FCS), 20 mM Hepes, 1000 IU/mL penicillin and 0.1 mg/mL streptomycin (all PAA, Coelbe, Germany). After cells had been isolated they were incubated at 37 °C with 8.5% CO_2_. Media changing was done every 3rd day until cells were confluent. For cell experiments cells were cultured in DMEM with 10% FCS and incubated at 37 °C with 5% CO_2_.

#### 4.4.2. GM7373

The endothelial cell line GM7373 was harvested from the aorta of a bovine calf. For cell culture, we used DMEM and 10% FCS. GM7373 was provided by Leibniz University, Institution of Biophysics, Hannover, Germany.

### 4.5. Cross Sections Established of Titanium Implants

Nine titanium scaffolds were seeded with 2.5 × 10^4^ GFP–osteoblasts (P 10). After one, three and seven days, three cell-seeded scaffolds each were fixed in 4% formalin. The samples were rinsed with tap water and dehydrated using an ethanol gradient (4 h in 70%, 80%, 90% and 100% ethanol). Afterwards, samples were defatted in acetone for 4 h, equilibrated with 100% ethanol for 6 h and embedded in methyl metacrylate for 2 days at 37 °C. Thirty-micrometer sections were cut with an internal hole saw (SP 1600, Leica, Wetzlar, Germany) and examined by fluorescence microscopy (DM4000B Leica microsystems, Wetzlar, Germany). Cell distance, cell amount, and difference in cell counting between upper and lower pores were examined by Wimasis Image Analysis (16–19 cross sections for each time point). The scale bar started at the scaffolds’ surface where cells had been settled and upper and lower pore were examined. Cells were counted as cell area, because after embedding the cells were unable to be counted separately. Only cells that had direct contact to the pores surface were counted. Cells that were not attached to the surface were ignored because they could be artifacts of the cutting process.

### 4.6. Migration Assays of GM7373 on HMGB1, VEGF, CXCL12

Twelve-well transwells (353182, BD Falcon, Erembodegem, Belgium) with 8 µm pore-size were used for the experiment. Seventy thousand GM7373 of passages 20 and 21 were added to a 37 °C pre-warmed 0.1% DMEM and incubated for 20 min. The following factors were added in duplicate: VEGF (450-32, Peprotech, Hamburg, Germany) (5/10/20 ng/mL), HMGB1 (H4652, Sigma–Aldrich, Taufkirchen, Germany) (50/100/150 ng/mL), CXCL12 (250-20A, Peprotech) (50/100/150 ng/mL), and VEGF combined with HMGB1 and HMGB1 plus CXCL12 using half of the mentioned dose. After that in duplicate two assays were performed using HMGB1 (50/100/150 ng/mL) plus CXCL12 (10/20/30 ng/mL) and HMGB1 (10/20/30 ng/mL) plus CXCL12 (50/100/150 ng/mL). Migration time was 4 h under cell culture conditions (37 °C, 5% CO_2_). Cells that did not migrate were removed with a cotton stick. Migrated cells in the lower chamber were trypsinated and counted with a cell counter (CellometerTM Auto 4, Nexcelom Bioscience, Lawrence, MA, USA).

### 4.7. Live Cell Imaging

LCI was performed with titanium implants and titanium PCL implants seeded with GFP–osteoblasts (LAS AF 2.6.0, Leica–Microsystems, Wetzlar, Germany).

Four titanium implants and three titanium PCL implants were placed in a 96 well plate filled with 150 µL DMEM and 10% FCS. GFP–osteoblasts (P 10) were added gently to the top of the scaffolds at a concentration of 2.5 × 10^4^ cells/150 μL medium. After a minimum 5 h incubation period at 37 °C, 5% CO_2_, the implants were turned upside down to visualize the cells in the inverse microscope. Subsequently, they were placed in new wells that were prepared with purpose-built Teflon constructs used for lifting the implants to create a gap between cells growing on the implants and the bottom of the culture plates for inverse microscopy. Then proliferation and motility of the cells could be observed by LCI Microscope (DMI 6000B Leica Microsystems, Wetzlar, Germany) during seven days with the program LAS AF 2.6.0. The same implant region was examined for seven days by taking pictures every 15 min. A constant temperature of 37 °C was kept and a 5% CO_2_ was ensured by using a heating unit and CO_2_ supplier. Cells of the same concentration without scaffolds were used for positive control. Cell count and cell spreading area were examined by Wimasis Image Analysis GmbH, Germany. The cell spreading area of osteoblasts was described by using the following equation: total cell area/total cell number.

### 4.8. Statistical Analysis

Statistical analyses were performed with SAS^®^ software, Version 9.3 (SAS Institute Inc., Cary, NC, USA). The significance level for all tests was set to *p* < 0.05. The data were shown as mean ± standard deviation.

## 5. Conclusions

The SLM generated titanium implants with 250-µm pore size enabled osteoblast seeding and proliferation into the deeper pore areas. PCL coating lowered the amount of adhered cell in comparison to the non-coated titanium structure. However, the cell number development and cell spreading area was unhindered by the PCL coating. Furthermore, the PCL coating method maintained the open porous structures. Thus, PCL can be used for implant coating and factor incorporation in structures ranging around 250 µm. HMGB1 showed even higher chomotactic potential on endothelial cells compared to VEGF *in vitro*. PCL coated porous titanium implants functionalized with HMGB1 would be a potent combination for supporting angiogenesis in bony defects.

## Supplementary Materials

Supplementary materials can be found at http://www.mdpi.com/1422-0067/16/04/7478/s1.
